# Exploring Oral Cavity Cancer in the United Arab Emirates (UAE)

**DOI:** 10.7759/cureus.53452

**Published:** 2024-02-02

**Authors:** Noura S AlNeyadi, Abdulrahman Bin Sumaida, Nandan M Shanbhag, Khalifa AlKaabi, Nouraddine A Alhasan, Syed Mansoor Hasnain, Omran El-Koha, Khalid Abdelgalil, Jawaher Ansari, Khalid Balaraj

**Affiliations:** 1 Otolaryngology - Head and Neck Surgery, Tawam Hospital, Al Ain, ARE; 2 Oncology/Radiation Oncology, Tawam Hospital, Al Ain, ARE; 3 College of Medicine and Health Sciences, United Arab Emirates University, Al Ain, ARE; 4 Oncology/Palliative Care, Tawam Hospital, Al Ain, ARE; 5 Radiation Oncology, Tawam Hospital, Al Ain, ARE; 6 Oncology, Tawam Hospital, Al Ain, ARE

**Keywords:** healthcare studies, treatment outcomes, oncology, clinical characteristics, retrospective analysis, united arab emirates, cancer epidemiology, oral cavity

## Abstract

Background

This study delves into the demographics and clinical characteristics of oral cavity tumors in the context of the United Arab Emirates. It further investigates the efficacy of four different treatment modalities in impacting patient survival rates. It aims to understand if any treatments significantly improve survival compared to others.

Methodology

To assess the survival outcomes across the different treatment groups, the study employed the log-rank test, a non-parametric statistical test widely used in survival analysis. The sample consisted of patients from the electronic medical records assigned to one of the following four treatment groups: radiotherapy only (RT), radiotherapy with surgery and chemotherapy (RT+S+C), radiotherapy with surgery (RT+S), and, finally, radiotherapy with chemotherapy including immunotherapy (RT+C). Data collection involved tracking survival times from the initiation of treatment until the last follow-up period or the occurrence of an event (e.g., death). The statistical analysis was conducted using the chi-squared statistic to determine the distribution of survival times across the groups, providing a quantitative measure of the difference between the observed and expected survival. The Kaplan-Meier curve was plotted for the cohort divided into four groups.

Results

The log-rank test yielded a p-value of 0.321019, suggesting no statistically significant difference in survival among the treatment groups at the 5% significance level. The chi-squared statistic was 3.498018, within the 95% acceptance region, further corroborating the null hypothesis of no significant survival difference across the groups. Despite this, an observed medium effect size of 0.59 indicates a moderate difference in survival between the groups.

Conclusions

The findings illustrate that while there is no statistically significant difference in survival rates among the four treatment groups, the medium effect size observed suggests a moderate difference in survival. This emphasizes the need to consider the statistical significance and effect size in clinical research, as they provide different insights into treatment efficacy.

## Introduction

Oral cavity cancers represent a significant and complex health concern globally. These cancers, predominantly squamous cell carcinomas, arise in the lips, tongue, gums, buccal mucosa, floor of the mouth, palate, and other parts of the oral cavity and pose considerable challenges in diagnosis and treatment. They account for about 3.6% of new cancer cases in the United States and have a five-year survival rate of 50% or less [1]. The most common histologic type of oral cavity cancer is squamous cell carcinoma, which is associated with risk factors such as tobacco, alcohol, and human papillomavirus infection [2,3]. Early detection and treatment of oral cavity cancers are crucial for improving survival and reducing morbidity. Still, many patients are diagnosed at advanced stages due to delays in seeking medical attention, referral, and diagnosis [4].

Oral cavity cancers are a significant public health concern in the Middle East and North Africa (MENA) region, where they are expected to double in incidence and mortality by 2030 [5]. Some of the common challenges faced by the MENA countries in the prevention and management of oral cavity cancers include lack of awareness, low screening rates, inadequate resources, and sociocultural barriers [6].

Oral cavity cancers, including those of the oropharynx, are aggressive and commonly invade local tissue, potentially leading to metastasis and high mortality rates. Despite improvements in treatment strategies, optimal results are yet to be achieved [7]. Traditional treatment options for oral cavity cancer have not significantly changed in decades, but advancements in disease management, surveillance, and reconstructive options have improved patient outcomes [8]. Of particular concern is the fact that these cancers are increasingly being diagnosed at a younger age, especially in individuals without traditional risk factors such as tobacco and alcohol use, indicating changing epidemiology [9]. The role of the oral microbiome in developing oral cancers has been a recent area of research, suggesting that chronic inflammation from periodontal diseases could be a contributing factor [10]. The incidence and risk factors for complications such as stroke post-surgery in oral cavity cancer patients, especially following neck dissection, have also been a subject of study, underscoring the complexity of treatment and recovery [11].

The incidence of oral cavity cancer significantly varies across the globe, with notable differences observed between developed and developing nations. This variation highlights the necessity for public health strategies tailored to specific regions. While the management and prognosis of oral cavity cancers have advanced over time, they have substantial effects on mortality, morbidity, and overall quality of life [12].

Understanding the causes and consequences of delays in treatment for oral cavity cancers is crucial. Timely treatment is not just a matter of clinical urgency it also has profound implications for patient outcomes and quality of life. A systematic review highlighted this aspect, identifying factors associated with delay and summarizing the effect of delay on oncological outcomes. The review found that non-Caucasian race, treatment in an academic setting, Medicaid/no insurance, and radiotherapy as primary treatment were associated with delays. Importantly, delays in the start of treatment for laryngeal and oral cavity cancer are linked to decreased overall survival despite the lack of a clear cut-off time point for these delays [13].

Furthermore, there is a pressing need for increased research and collaborative efforts, especially among countries in the MENA region. Developing and implementing effective strategies for controlling and caring for oral cancer in these regions is vital and can be achieved by fostering collaboration and focusing on region-specific strategies, thus improving the outcomes and quality of life.

## Materials and methods

This retrospective cohort study was conducted at Tawam Hospital, a leading tertiary care center in the United Arab Emirates (UAE). The study conducted a comprehensive review of medical records of patients diagnosed with oral cavity cancer from January 2012 to December 2023. This period provided a substantial temporal frame to observe long-term trends and outcomes.

The study population comprised patients diagnosed with oral cavity cancer, as confirmed by histopathological examination. Inclusion criteria were (1) diagnosis of oral cavity cancer, (2) age 18 years or older at the time of diagnosis, and (3) availability of complete medical records. Patients were excluded if they had a history of other primary malignancies or incomplete data.

Data were extracted from electronic medical records, encompassing demographic details (age, gender, nationality), clinical characteristics (performance status, presence of comorbidities such as diabetes mellitus, hypertension, dyslipidemia), lifestyle factors (smoking, alcohol consumption), and detailed cancer-specific information (date of diagnosis, histopathology, tumor differentiation, stage at diagnosis, laterality). Treatment data included surgery, chemotherapy, radiation therapy, immunotherapy, and specific regimens. Follow-up data were also recorded, including patient outcomes (alive or deceased), date of last follow-up, and overall survival time in months.

Descriptive statistics were used to summarize the demographic and clinical characteristics of the cohort. Categorical variables were presented as frequencies and percentages, while continuous variables were summarized using means, standard deviations, medians, and interquartile ranges, depending on their distribution. Inferential statistical analyses were conducted to explore associations between various factors and patient outcomes. Survival analysis, including Kaplan-Meier curves and Cox proportional hazards models, was utilized to assess factors influencing survival. All statistical analyses were performed using Microsoft Excel®, RStudio, and/or Python with a significance level set at p-values <0.05. The Kaplan-Meier curve was plotted for the cohort divided into the following four groups: radiotherapy only (RT), radiotherapy with surgery and chemotherapy (RT+S+C), radiotherapy with surgery (RT+S), and, finally, radiotherapy with chemotherapy including immunotherapy (RT+C).

This study was conducted following the Declaration of Helsinki guidelines and was approved by the Institutional Review Board of the Tawam Human Research Ethics Committee (approval number: MF2058-2024-1022). Given the study’s retrospective nature, patient consent was waived, but strict confidentiality and anonymity of patient data were maintained throughout the research process.

## Results

This retrospective analysis encompassed a cohort of 44 patients, ranging in age from 31 to 88 years (mean age of 54.5 ± 12.43 years). This cohort was characterized by a notable male predominance, with 33 (75%) male patients, and a significant proportion of non-national patients, comprising 95.45% (42 patients) of the study population. The Eastern Cooperative Oncology Group (ECOG) performance status, which reflects the patient’s ability to perform daily activities, revealed that the majority of the cohort, 65.85% (27 patients), had a score of 0. This indicates that most patients were functionally independent at the time of diagnosis or initiation of treatment.

Regarding comorbid conditions, the prevalence of diabetes mellitus and hypertension was relatively comparable within the cohort. Approximately one-third of the patients were diagnosed with diabetes mellitus (31.82%, 14 patients), and just over one-third had hypertension (34.09%, 15 patients). Of note, the majority of patients did not present with dyslipidemia. Additionally, lifestyle factors such as smoking were nearly equally distributed among the cohort, underscoring the potential influence of lifestyle choices on health outcomes. Most patients had undergone neck radiation (81.82%, 36 patients) and chemotherapy (70.45%, 31 patients). Conversely, a smaller segment of the cohort received immunotherapy (20.45%, 9 patients) and platinum-based therapies (63.64%, 28 patients) (Table 1).

**Table 1:** Demographic and clinical characteristics of the patient population (N = 44). Data are represented as n (%). Platinum indicates those who received platinum chemotherapy. DM = diabetes mellitus; HTN = hypertension

Gender	Male	Female
	33 (75%)	11 (25%)
Nationality	National	Non-national
	2 (4.55%)	42 (95.45%)
Variable	Yes	No
DM	14 (31.82%)	30 (68.18%)
HTN	15 (34.09%)	29 (65.19%)
Dyslipidemia	6 (13.64%)	38 (86.36%)
Smoking	23 (52.27%)	21 (47.73%)
Neck radiation	36 (81.82%)	8 (18.18%)
Chemotherapy	31 (7.45%)	13 (29.55%)
Immunotherapy	9 (20.45%)	35 (79.55%)
Platinum	28 (63.64%)	16 (36.36%)
Surgery	31 (7.45%)	13 (29.55%)

**Figure 1:**
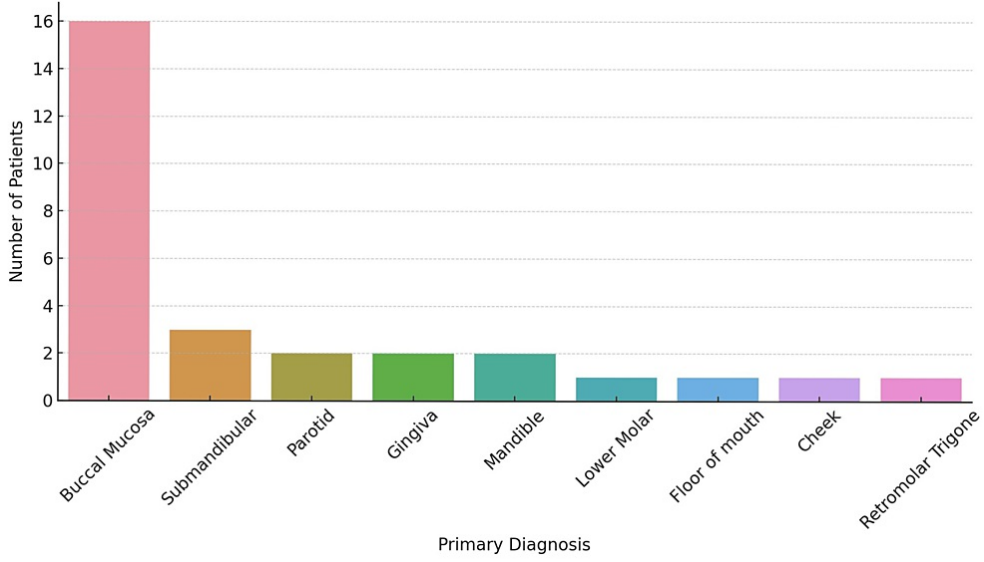
Primary site (N = 44). Data are represented as the number of patients along the y-axis and the primary site within the oral cavity along the x-axis.

The patient outcomes demonstrated a survival rate of 77.27%, emphasizing the effectiveness of the treatments or the early stage of disease detection in this specific cohort. The buccal mucosa was the primary site with the most involvement (Figure 1).

**Figure 2:**
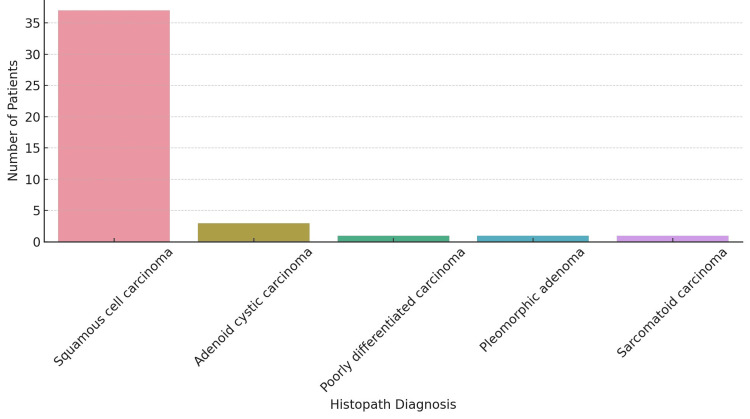
Histopathology (N = 44). The figure depicts the histopathology along the x-axis and the number of patients along the y-axis.

Squamous cell carcinoma was the most common histopathological diagnosis (Figure 2).

**Figure 3:**
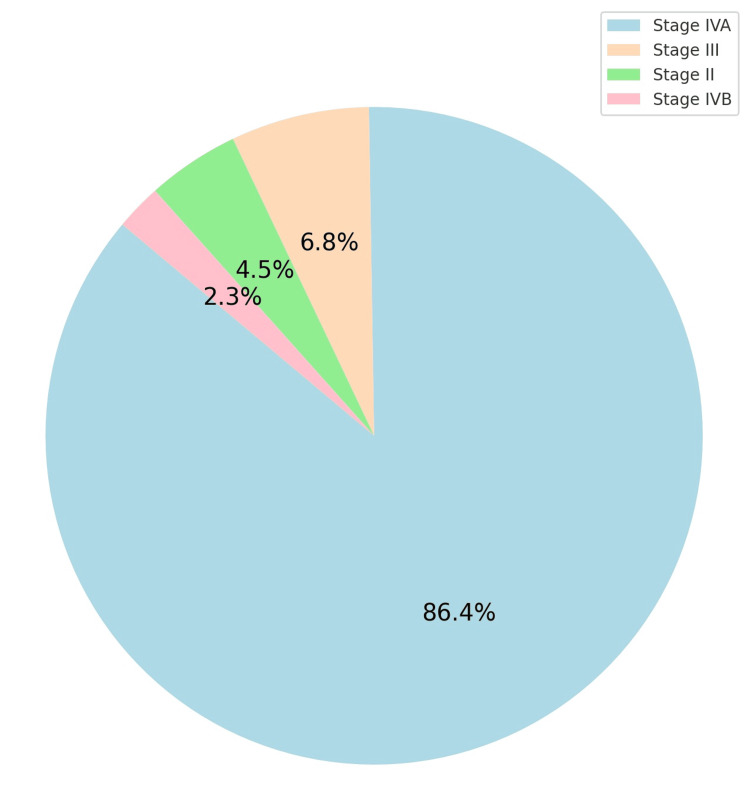
Stage of the cancer (N = 44). Staging as per the American Joint Commission for Cancer Staging (AJCC): light blue = Stage IVA (38 patients); peach: Stage III (3 patients); light green: Stage II (2 patients); pink: Stage IVB (1 patient)

Most patients presented with locally advanced cancer (Figure 3).

**Figure 4:**
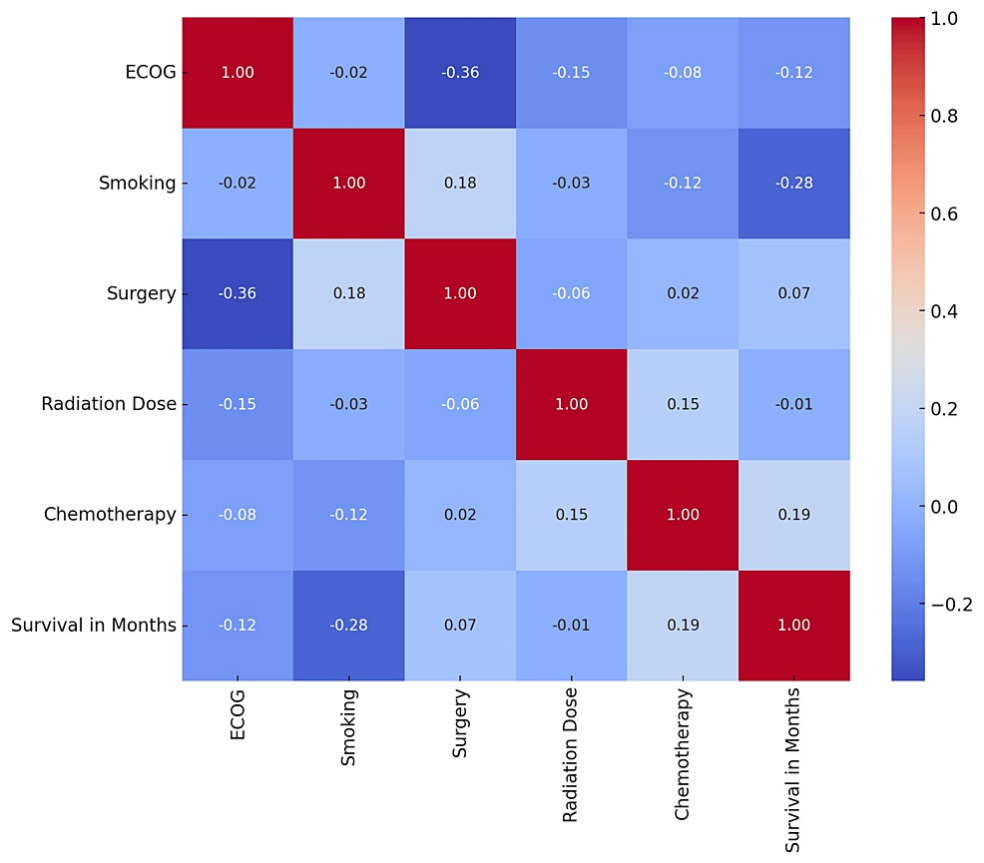
Correlation heat map between variables. ECOG = Eastern Cooperative Oncology Group performance status

A correlation heatmap was generated to understand the relationship between smoking, ECOG performance status, radiation dose, chemotherapy, surgery, and survival in months (Figure 4). The interpretation is discussed in the Discussion section.

**Figure 5:**
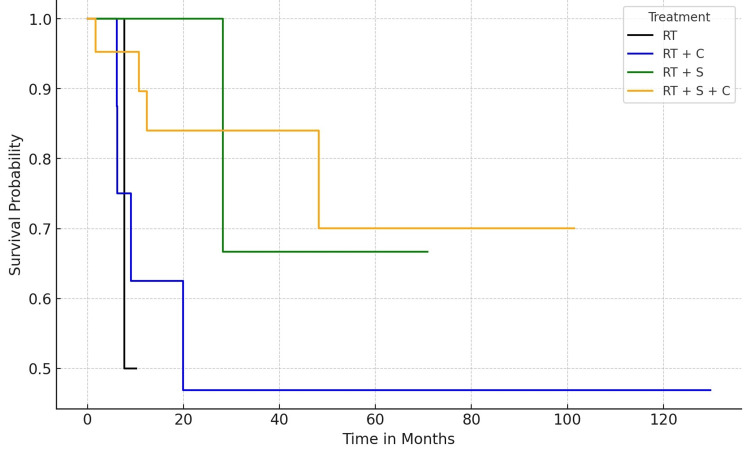
Survival probability of the four groups. RT (black) = radiation only; RT+C (blue) = radiotherapy and systemic therapy (chemotherapy and immunotherapy); RT+S (green) = radiotherapy and surgery; RT+S+C (orange) = radiotherapy, surgery and systemic therapy

Finally, the cohort was divided into the following four groups: RT - those who received radiotherapy only, RT+S - those who underwent surgery and radiotherapy, RT+C - those treated with systemic therapy (chemotherapy+immunotherapy) and radiotherapy, and, finally, RT+S+C - those who underwent surgery, followed by radiotherapy and chemotherapy. A Kaplan-Meier curve for survival in months was plotted for these groups (Figure 5).

The *at-risk* number and the *event *are provided in Table 2.

**Table 2:** Number of patients at risk and the events (N = 40). The data are represented as n (%). RT = radiation only; RT+C = radiotherapy and systemic therapy (chemotherapy and immunotherapy); RT+S = radiotherapy and surgery; RT+S+C = radiotherapy, surgery and systemic therapy

Time in months	0	25	50	75	100
At risk					
RT	4 (10%)	0	0	0	0
RT+C	8 (20%)	3 (7.5%)	2 (5%)	1 (2.5%)	1 (2.5%)
RT+S	6 (15%)	3 (7.5%)	1 (2.5%)	0	0
RT+S+C	22 (55%)	11 (27.5%)	5 (12.5%)	2 (5%)	1 (2.5%)
Events					
RT	0	1 (2.5%)	1 (2.5%)	1 (2.5%)	1 (2.5%)
RT+C	0	4 (10%)	4 (10%)	4 (10%)	4 (10%)
RT+S	0	0	1 (2.5%)	1 (2.5%)	1 (2.5%)
RT+S+C	0	3 (7.5%)	4 (10%)	4 (10%)	4 (10%)

The log-rank test assessed the differences in survival distributions among the four treatment groups. The resulting p-value was 0.321019, demonstrating no statistically significant difference in survival between the groups when considering a threshold of p < 0.05 for statistical significance. The chi-squared statistic was 3.498018, which falls within the 95% acceptance region, further supporting the null hypothesis of no significant survival difference. Despite the lack of statistical significance, the observed medium effect size (0.59) suggests a moderate difference in survival between the groups.

## Discussion

This retrospective analysis of cancer patients presents critical insights into patient demographics, comorbidities, treatment modalities, and outcomes. The age range and mean age of the patients in this cohort are indicative of cancer’s prevalence in middle-aged and older populations. This aligns with existing research that suggests age is a significant factor in cancer incidence and outcomes, with older age groups often presenting more complex treatment challenges due to comorbidities and general health status [14]. The predominance of males in the cohort, 75% (33 patients) of the patients, is consistent with trends observed in other studies, where certain types of cancers are more common in males. However, this also raises questions about gender-based biological, environmental, and lifestyle factors that might influence cancer prevalence and outcomes [15]. The study’s high percentage of non-national patients is a unique aspect that could reflect demographic patterns specific to the study region or indicate broader global cancer epidemiology trends. This emphasizes the need for culturally sensitive and accessible healthcare services, especially in oncology [16]. This predominantly male and non-national cohort aligns with the findings of other studies highlighting the demographic variations of the cancer population [17].

The ECOG performance status is a critical measure in this study, with a majority scoring of 0. This suggests many patients were functionally independent at diagnosis or treatment initiation, often associated with better treatment outcomes and higher survival rates [18]. The presence of comorbidities such as diabetes and hypertension in about a third of the patients is consistent with the growing recognition of the impact of such comorbidities on cancer outcomes [19]. Notably, the majority not having dyslipidemia could suggest a lesser role of this particular comorbidity in the studied cancer type, though this requires further investigation.

The correlation heatmap revealed several insights, most significantly a negative correlation between ECOG and survival in months. This suggests that higher ECOG scores (worse physical condition) are associated with shorter survival times, which aligns with clinical expectations [20,21]. Smoking shows a weak correlation with other variables, which again reiterates the detrimental effect of smoking on survival in cancers, in general, and head and neck squamous cell carcinomas, in particular [22]. Similarly, the correlation between cancer stage and survival is negative, indicating that patients with advanced cancer stages tend to have shorter survival times. This is consistent with medical knowledge, as advanced-stage cancers are often more aggressive and more challenging to treat [23,24].

Both surgery and chemotherapy show a positive correlation with survival, suggesting that patients who underwent these treatments had longer survival times. This could indicate the effectiveness of these treatments in prolonging life, though it is important to remember that correlation does not imply causation. The high prevalence of neck radiation and chemotherapy in the cohort indicates an aggressive treatment approach, which is often seen in more advanced stages of cancer [25].

The survival analysis focused on comparing the outcomes of four distinct treatment groups, namely, RT, RT+S, RT+C, and RT+S+C. The study evaluated the survival probabilities and cumulative events (deaths) for up to 100 months. The Kaplan-Meier survival curves, plotted for each treatment group, revealed distinct patterns in survival probabilities over time. The RT group, treated solely with radiotherapy, exhibited a gradual decline in survival probability, indicative of a steady rate of events over the 100 months. In contrast, the RT+C group initially showed a more pronounced decrease in survival probability, suggesting a higher event rate in the early months, which stabilized over time. The survival curve of the RT+S group suggested a similar pattern to the RT group but with slightly better survival probabilities, potentially indicating a beneficial effect of adding surgery to radiotherapy. The most noteworthy observation was in the RT+S+C group, which demonstrated a more favorable survival probability than other groups, especially in the early and middle phases of the timeline. This suggests a potential synergistic effect of combining all three treatments.

The cumulative event analysis further elucidated the impact of different treatments. At the initial 25-month mark, the RT+C group had the highest number of events (four deaths), while the RT and RT+S groups had fewer events (one death each). The RT+S+C group also experienced three events, indicating an initial phase of higher mortality, which stabilized afterward. Interestingly, by the 50-month interval, the cumulative events in the RT+S+C group increased to four, equaling the RT+C group, but did not increase further up to the 100-month mark. This suggests an initial period of vulnerability followed by a stabilization in the RT+S+C group.

The survival analysis suggests combining radiotherapy, surgery, and chemotherapy (RT+S+C) may offer a survival benefit, particularly in the treatment’s initial and middle phases. However, this benefit appears to plateau over time. The initial higher mortality in the RT+C and RT+S+C groups might be attributed to the aggressive nature of combined treatments or more advanced stages of disease at the onset of treatment. While surgery remains the cornerstone of the management of locally advanced oral cavity cancers, postoperative radiotherapy is often utilized in the majority of cases in this cohort, patients with more advanced disease characteristics, including pT3-4 stages, close surgical margins, and N2/3 nodal involvement. Additionally, the presence of disease in level IV-V lymph nodes, perineural invasion, and lymphovascular space invasion were noted as significant factors for radiotherapy in line with the literature [26,27]. Extracapsular extension and/or positive margins formed the basis for combining all three modalities (RT+C+S) [28,29].

In the context of oral cavity cancers, advanced stages and treatment resistance often lead to higher recurrence rates and poorer survival. However, introducing precision-targeted therapies such as stereotactic radiosurgery, hypofractionated stereotactic radiation, and brachytherapy in the UAE adds promise in managing small-volume recurrences [30].

Considering these findings in the context of the disease’s natural history and the potential side effects of combined treatments is essential. Further research is warranted to understand the long-term outcomes and quality of life implications for patients undergoing these treatment modalities.

Limitations

The study’s reliance on retrospective data from a single healthcare center may not accurately represent the broader oral cavity cancer patient population, limiting the generalizability of the findings. The predominance of male and non-national patients in the study cohort might not reflect the diversity of the oral cavity cancer population, potentially influencing the applicability of the results to different demographic groups. The small sample size in the study does not isolate the individual effects of each treatment modality (radiotherapy, surgery, chemotherapy) adequately, making it challenging to determine the specific contribution of each to patient survival and overall treatment efficacy.

## Conclusions

This study provides valuable insights into the demographics, treatment modalities, and outcomes of oral cavity cancer patients in the UAE, highlighting the complexity and challenges in managing this disease. The findings showcase the importance of considering patient-specific factors, such as age, gender, nationality, and comorbidities, in tailoring treatment approaches. The observed correlations between treatment modalities and survival outcomes offer promising directions for future research, particularly in exploring the synergistic effects of combined treatments.

However, the limitations noted above, including the study’s retrospective nature, demographic heterogeneity, and challenges in assessing individual treatment efficacy, necessitate caution in interpreting the results. Future studies should aim to address these limitations, possibly through prospective, multicenter trials with more diverse patient populations. Additionally, there is a need for more in-depth research into the long-term outcomes and quality of life implications of various treatment combinations to optimize care for oral cavity cancer patients.

Overall, while the study contributes to our understanding of oral cavity cancer treatment, it also highlights the need for ongoing research to refine treatment strategies and improve patient outcomes in this challenging field.
